# Mitochondrial DNA Parameters in Blood of Infants Receiving Lopinavir/Ritonavir or Lamivudine Prophylaxis to Prevent Breastfeeding Transmission of HIV-1

**DOI:** 10.3390/jcm9092972

**Published:** 2020-09-14

**Authors:** Audrey Monnin, Nicolas Nagot, Marianne Periès, Roselyne Vallo, Nicolas Meda, Mandisa Singata-Madliki, James K. Tumwine, Chipepo Kankasa, Nobubelo Ngandu, Ameena Goga, Pascal Reynier, Thorkild Tylleskär, Philippe Van de Perre, Jean-Pierre Molès

**Affiliations:** 1 Pathogenèse et Contrôle des Infections Chroniques, INSERM U1058, Université Montpellier, Etablissement Français du Sang, 34934 Montpellier, France; marianne.peries@umontpellier.fr (M.P.); roselyne.vallo@umontpellier.fr (R.V.); jean-pierre.moles@inserm.fr (J.-P.M.); 2 Pathogenèse et Contrôle des Infections Chroniques, INSERM U1058, Université Montpellier, Centre Hospitalier Universitaire, 34934 Montpellier, France; n-nagot@chu-montpellier.fr (N.N.); p-van_de_perre@chu-montpellier.fr (P.V.d.P.); 3 Centre MURAZ, Bobo-Dioulasso 01 B.P. 390, Burkina Faso; nicolas.meda@gmail.com; 4 Effective Care Research Unit, Cecilia Makiwane Hospital, University of Fort Hare, East London 5201, South Africa; mandisa.singata@gmail.com; 5 Department of Paediatrics and Child Health, School of Medicine, College of Health Sciences, Makerere University, Kampala 7062, Uganda; kabaleimc@gmail.com; 6 Department of Paediatric and Child Health, University Teaching Hospital, Lusaka PO Box 50110, Zambia; ckankasa@zamnet.zm; 7 Health Systems Research Unit, South African Medical Research Council, Cape Town 7501, South Africa; nobubelo.ngandu@mrc.ac.za (N.N.); ameena.goga@mrc.ac.za (A.G.); 8 Département de Biochimie et Génétique, Centre Hospitalier Universitaire, 49933 Angers, France; pareynier@chu-angers.fr; 9 Centre for International Health, Faculty of Medicine, University of Bergen, 5009 Bergen, Norway; thorkild.tylleskar@uib.no

**Keywords:** mitochondrial DNA, depletion, deletion, HIV-exposed uninfected children, lopinavir/ritonavir, lamivudine, breastfeeding, Africa

## Abstract

Children who are human immunodeficiency virus (HIV)-exposed but uninfected (CHEU) accumulate maternal HIV and antiretroviral exposures through pregnancy, postnatal prophylaxis, and breastfeeding. Here, we compared the dynamics of mitochondrial DNA (mtDNA) parameters in African breastfed CHEU receiving lopinavir/ritonavir (LPV/r) or lamivudine (3TC) pre-exposure prophylaxis during the first year of life. The number of mtDNA copies per cell (MCN) and the proportion of deleted mtDNA (MDD) were assessed at day 7 and at week 50 post-delivery (PrEP group). mtDNA depletion was defined as a 50% or more decrease from the initial value, and mtDNA deletions was the detection of mtDNA molecules with large DNA fragment loss. We also performed a sub-analysis with CHEU who did not receive a prophylactic treatment in South Africa (control group). From day seven to week 50, MCN decreased with a median of 41.7% (interquartile range, IQR: 12.1; 64.4) in the PrEP group. The proportion of children with mtDNA depletion was not significantly different between the two prophylactic regimens. Poisson regressions showed that LPV/r and 3TC were associated with mtDNA depletion (reference: control group; LPV/r: PR = 1.75 (CI95%: 1.15–2.68), *p* < 0.01; 3TC: PR = 1.54 (CI95%: 1.00–2.37), *p* = 0.05). Moreover, the proportion of children with MDD was unexpectedly high before randomisation in both groups. Long-term health impacts of these mitochondrial DNA parameters should be investigated further for both CHEU and HIV-infected children receiving LPV/r- or 3TC- based regimens.

## 1. Introduction

The most recent Word Health Organisation (WHO) guidelines for the prevention of mother-to-child transmission (PMTCT) of human immunodeficiency virus 1 (HIV-1) recommend lifelong antiretroviral therapy (ART) for all pregnant or breastfeeding HIV-infected women independently of their CD4 count or HIV clinical stage [1]. In addition to in utero exposure to both HIV and the antiretroviral drugs (ARVs), children who are HIV-exposed but uninfected (CHEU) continue to be exposed to HIV and the ARVs during breastfeeding as well as for 6–12 weeks of recommended pre-exposure prophylaxis (PrEP) with either nevirapine (NVP) alone or in combination with zidovudine (ZDV) [1]. Furthermore, maternal ART results in significant ARV drug concentrations in breast milk to which the baby is also exposed for several months [2,3,4,5]. In 2019, 15.2 million children were CHEU worldwide, thus exposed to HIV and ARV [6]. Many unfavourable health outcomes have been reported in CHEU such as metabolic disorders [7,8], increased infectious disease morbidity and higher mortality [9,10,11,12], impaired growth [13,14,15,16,17,18], neurodevelopmental delays [17,19,20,21], altered immunity [10,22,23,24,25,26], and mitochondrial toxicity in comparison to never HIV-exposed children [27,28,29,30]. Taken together, this prompted Powis and Siberry to advocate for the long-term follow up of CHEU irrespective of their HIV/ARV exposures [31].

Reduction of mitochondrial DNA (mtDNA) copy number per cell (MCN) is a well-known side effect of nucleoside reverse transcriptase inhibitors (NRTIs) [32,33]. NRTIs can act as chain terminators either by incorporation into the mtDNA which leads to an aborted replication, or by directly inhibiting DNA polymerase γ, the enzyme responsible for mtDNA replication [32,34]. Depending on the nature of the different NRTI drugs, different degrees of inhibition have been estimated with the following decreasing drug toxicity scheme: zalcitabine ≥ didanosine ≥ stavudine >> lamivudine (3TC) > tenofovir > emtricitabine ≥ zidovudine > abacavir [32]. As a consequence of the decrease of MCN, the cellular oxidative phosphorylation is disturbed, which leads to increased production of reactive oxygen species (ROS) that in turn may generate mtDNA point mutations and/or deleted mtDNA (MDD) [32,34]. Recent in vitro studies showed that most of the protease inhibitors (PIs) can induce mitochondrial-induced apoptosis [35,36,37] and increase ROS production [36,37,38,39,40]. These two mechanisms can lead to a decrease of mtDNA content and induce mtDNA mutations, as NRTIs do. Furthermore, LPV-boosted RTV (LPV/r) showed to be the most potent inducer of ROS among the other tested PIs [38]. In this regard, mtDNA depletion has been reported in HIV-infected children receiving ART compared to their healthy counterparts [41,42,43]. CHEU exposed in utero to ARVs, and particularly to NRTIs, also have lower MCN at birth compared to uninfected children, ranging from 37.0% to 49.0% in cord blood leukocytes [44,45,46,47], endothelial cord blood cells [45,46], and PBMCs, which persisted until two years of age with a progressive reversion to baseline mtDNA levels around five years of age [48]. In contrast, several other studies have reported higher MCN at birth [49,50,51,52], and in one of them, a longitudinal follow-up showed a decrease over time [52]. In addition, like other cellular DNA, mtDNA is susceptible to acquired mutations [53], and the accumulation of deleted mtDNA (MDD) is part of the ageing process [53,54]. It is noteworthy that mtDNA deletions have rarely been investigated in CHEU and an association with ARV drug exposure has never been demonstrated [55,56].

Our study aimed at evaluating the acute mitochondrial toxicity of an extended PMTCT prophylaxis based on 3TC or LPV/r regimen given to CHEU for one year by analysing mtDNA parameters, i.e., MCN and MDD.

## 2. Experimental Section

### 2.1. Design and Study Population

In this longitudinal study, we included CHEU already enrolled in the ANRS 12,174 trial (NCT00640263), referred hereafter as the PrEP group in the main analysis. Samples were collected between November 2009 and May 2012 in four African countries namely Burkina Faso, South Africa, Uganda, and Zambia. The trial protocol and the main findings have been published elsewhere [57,58]. In brief, HIV-infected mothers that enrolled in the trial received ARV prophylaxis during pregnancy and labour as per national PMTCT guidelines at the time of the trial [59]. They had a CD4 cell count above 350 cells/mm3 and were therefore not eligible for ART at that time [59]. Children received 7 days of nevirapine as post-partum prophylaxis. Seven days after birth (D7) and subsequently at one-month intervals, children were screened for HIV infection by HIV DNA polymerase chain reaction (PCR). HIV-negative children were included in the trial and randomized to receive either LPV/r or 3TC during breastfeeding (maximum 50 weeks, W50). Prophylaxis was discontinued one week after the cessation of breastfeeding, or at W50 (maximum duration). 

Criteria for 1236 CHEU included: (1) provision of informed consent to store and use samples for future research, (2) completion of the W50 visit, (3) availability of dried blood spots (DBS) for the D7 and W50 visits and, (4) not HIV-infected at W50. Among these eligible participants, 200 CHEU were randomly chosen in each of the three trial sites (Ouagadougou, Burkina Faso; Mbale, Uganda and Lusaka, Zambia). For the fourth site (East London, Eastern Cape province, South Africa), 50 children were randomly chosen from a restricted list, created for a sub-study on in-depth adherence evaluation and treatment acceptability. The gender ratio of the participants was roughly 1:1 (Male:Female). Socio-demographic, biological, clinical, and infant feeding characteristics for all mothers and children were obtained from the ANRS 12174 trial database. Maternal CD4 cell count was measured before delivery and plasma HIV viral load at 38 weeks (W38) post-partum. Plasma HIV RNA under 1000 copies/mL was considered as a controlled viral load [1]. 

We also included a control group of 40 South African CHEU from the South African prevention of mother-to-child transmission evaluation survey (recruitment from September 2012 to March 2013) [60]. This national program enrolled CHEU aged of 4–8 weeks, attending clinics for their first immunization visit. Although WHO guidelines at that time recommended maternal ARV prophylaxis from 14 weeks of gestation until 7 days after giving birth and infant prophylaxis for 6 weeks or until one week after cessation of breastfeeding (WHO Option A), these 40 CHEU had not been exposed to any maternal or infant ARV prophylaxis [60]. Indeed, mothers of these children did not know their HIV positive status, thus they had not received ARV prophylaxis, nor had their children. These mothers were identified because their babies tested HIV antibody positive. This group of CHEU was referred to the control group in a sub-analysis restricted to South African children. These CHEU came from eight provinces of South Africa (Eastern Cape, Free Sate, Gauteng, Kwazulu Natal, Mpumalanga, Northern Cape, and Northern West and Western Cape). Samples were collected at six weeks of age (W6) because samples at D7 were not available, and at W26 and W50.

The primary outcomes were the proportion of children with mitochondrial DNA (mtDNA) depletion at W50 (PrEP group) or W26 (control group) and the proportion of children with mtDNA deletions at D7 and W50 (PrEP group) or at W6 and W26 (control group).

### 2.2. Sample Collection and Processing 

Capillary blood samples were collected by heel prick on Whatman 903 cards (DBS) and stored at −20 °C at the study sites. DNA extraction was performed from 3-mm diameter punches (*n* = 3) using the QIAamp DNA Blood Mini Kit (Qiagen, Hilden, Germany) following the manufacturers’ instructions. Extracted DNA were stored at −80 °C. 

### 2.3. Mitochondrial DNA Parameters Assays

#### 2.3.1. Mitochondrial DNA Copy Number Per Cell

Blood mtDNA content was assessed using the QuickScanTM Mitox assay (Primagen, Amsterdam, Netherlands) [61]. mtDNA (RNR2 gene; forward 5′-GGGCTCTGCCATCTTAA-3′ and reverse 5′-GTAATCCAGGTCGGTTTCTA-3′) and nuclear DNA (snRNP U1A gene; forward 5′-CGGCATGTGGTGCATAA-3′ and reverse 5′-TGCGCCTCTTTCTGGGTGTT-3′) were amplified by quantitative PCR (qPCR) on extracted DNA and quantified using calibrators provided by the manufacturer. Results were given as mtDNA copy number per cell (MCN) using Equation (1) (according to the manufacturer instructions):

(1) $$\mathrm{MCN}\;=\;2^{(\left[Ct\left(A\right)*E\left(A\right)\right]-\left[Ct\left(B\right)*E\left(B\right)\right]},$$

where *Ct* is Cycle threshold, *E* is efficacy of the PCR, and *A* and *B* are the genes of interest, respectively, mtDNA and U1A (see the Methodology details section in the Supplementary Materials for descriptive statistics of the assay).

As a quality control, we used a modified analysis flow described by Ashar et al. to check for nuclear DNA degradation. At all points in time (D7, W6, W26, and W50 in the PrEP and control groups), if a cycle threshold (Ct) for U1A was not comprised between the mean of all the *Ct* ± (2 × standard deviation), the sample was removed from the analysis [62]. 

Because we quantified MCN in whole blood, we also performed correlation analyses for each site at all points in time between platelet count and MCN using Spearman’s rank-order correlation. A site was excluded from the analysis if the absolute value of Rho was ≥0.3 with *p* ≤ 0.05. We further controlled for the mitochondria-rich platelet content using platelets and leucocytes enumerated on the MCN values at D7 by applying the formula proposed by Hurtado et al. [63]. We assumed that raw data were validated if the difference between them and corrected data did not exceed 10%.

The decrease in MCN between the MCN measured at *t0* (D7 or W6) and the one measured at *t*1 (W26 or W50) was calculated according to Equation (2):

(2) $$\frac{\left(MCN\;at\;t0\right)-\left(MCN\;at\;t1\right)}{MCN\;at\;t0}\times 100,$$

We defined mtDNA depletion as a 50% or more decrease in MCN from D7 to W50 for the PrEP group or from W6 to W26 for the control group. No given threshold for mtDNA depletion is described in the literature and a few longitudinal studies have reported conflicting results. Indeed, reports from birth to one year of age in healthy US children have shown no variation of MCN in leucocytes [48], increased MCN in leucocytes [44], or decreases of MCN by 29% in PBMC [52]. Another Chinese study showed a decreasing trend of MCN during the first two years of life [64]. For CHEU, US studies have reported a decrease of MCN by 5.3% in PBMC [52], or an increase of MCN in leucocytes [44]. As a conservative threshold, a 50% reduction encompassed all of these reported values.

#### 2.3.2. Mitochondrial DNA Deletion

Primers MITO which target a mtDNA region where no deletion has been reported (forward 5′-CTAAATAGCCCACACGTTCCC-3′ and reverse 5′-AGAGCTCCCGTGAGTGGTTA-3′) and primers DEL which target a region that encompasses 84% of the reported deletions so far (forward 5′-CTGTTCCCCAACCTTTTCCT-3′ and reverse 5′-CCATGATTGTGAGGGGTAGG-3′) were used in a duplex qPCR as previously described [65]. qPCRs were performed on a LightCycler^®^ 480-II System (Roche, Bâle, Switzerland) using the following cycling program: 95 °C for 10 min; 40 cycles at 95 °C for 15s, 55 °C for 15 s, and 60 °C for 1 min. The final reaction volume was 25 µL and contained 10 µL LightCycler^®^480 Probes Master mix (Roche, Bâle, Switzerland), each mitochondrial DNA primer at 500 nM and their respective probes at 50 nM and 150 nM for MITO and DEL, respectively. Results were obtained as a proportion of deleted mtDNA (MDD). 

We considered children as being with MDD when the proportion of deleted mtDNA was above 2.3% of the total mtDNA. This value was determined as the mean value obtained for 10 healthy newborns + 3 standard deviations. Because we did not have enough samples with a known percentage of deletion to test for the proportionality of this assay, we used the results in a qualitative manner. 

Both qPCRs were validated by DNA samples provided by a reference center for mitochondrial diseases from Angers, France (see the Appendix A and Appendix B for methodology details related to mitochondrial DNA parameters assays and Supplementary Materials Figures S1 and S2 for descriptive statistics of the deletion assay and results details for the quality control of DNA).

**Figure 1:**
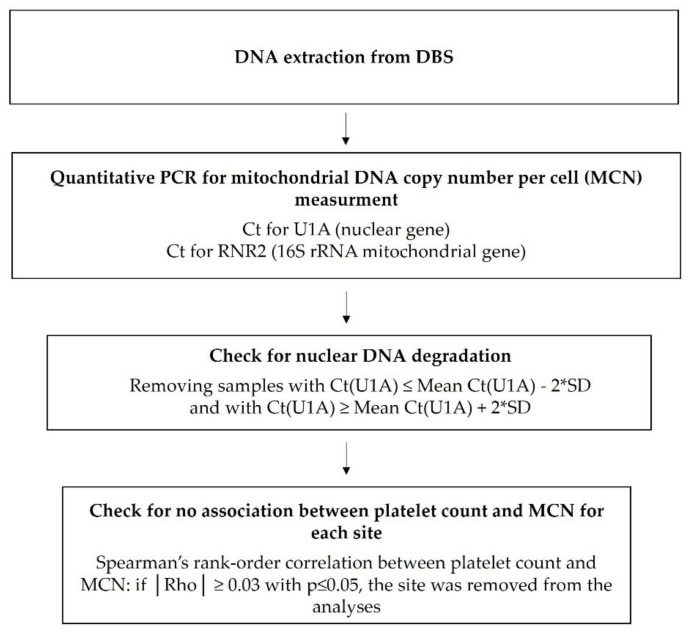
Analysis flow for mitochondrial DNA copy number per cell assessment. Abbreviations: DBS, dried blood spot; PCR, polymerase chain reaction; Ct, cycle threshold; SD standard deviation.

The analysis flow for MCN measurement is summarised in Figure 1.

### 2.4. Statistical Analysis

The data were described as frequencies with percentages for categorical variables, and medians with interquartile ranges (IQR) or means with standard deviations (SD) for non-Gaussian or Gaussian continuous variables, respectively. Prophylactic treatment comparisons were performed using Chi square or Fisher’s exact tests for categorical variables and Wilcoxon Mann–Whitney test for non-Gaussian continuous variables or Student’s t-test for Gaussian continuous variables. 

Mitochondrial DNA (mtDNA) copy number per cell (MCN) at baseline was compared between sites using Kruskal–Wallis test because the variable did not fulfil the normality condition. Wilcoxon Mann–Whitney test was used for paired comparisons. MCN between genders at baseline was compared using Wilcoxon Mann–Whitney test. We performed a Spearman’s rank correlation test to address whether there was a correlation between HIV-related maternal characteristics at baseline and MCN at D7.

In order to evaluate the impact of 3TC and LPV/r on mtDNA, both mtDNA depletion and mtDNA deletion were assessed. Prophylactic treatments comparison was performed using Wilcoxon Mann-Whitney test and Chi square test. In order to address whether the prophylaxis was associated with mtDNA depletion, we used Poisson regression with robust error variance to model the probability of having a mtDNA depletion (≥50% reduction of MCN) at W26 in a sub-analysis restricted to the South African site including CHEU children who did not received a postnatal prophylactic treatment (control group). Variables with *p* ≤ 0.25 in the bivariate analysis were included in the multivariable analysis. Variables were then selected according to a backward elimination for minimizing the Akaike information criterion (AIC). The confidence level was set at 95% and power at 80%. The same plan of analysis was used in order to characterise the impact of each drug on mtDNA depletion in this sub-analysis. Statistical analyses were performed using SAS studio (Copyright © 2012–2016, SAS Institute Inc., Cary, NC, USA).

### 2.5. Ethics Considerations

Written informed consents were obtained from the mother or legal representative prior to enrolment in the ANRS 12,174 trial (NCT00640263). The protocol was conducted in accordance with the Declaration of Helsinki and approved by the Biomedical Research Ethics Committee in Zambia (No 008-02-080), the Uganda National Council for Science and Technology (No HS 470), the Stellenbosh University ethics committees for South Africa (No M09/11/043) and the Ethical Committee for Health Research in Burkina Faso (No 2008-039). 

## 3. Results

### 3.1. Study Population Characteristics of the PrEP Group

**Figure 2:**
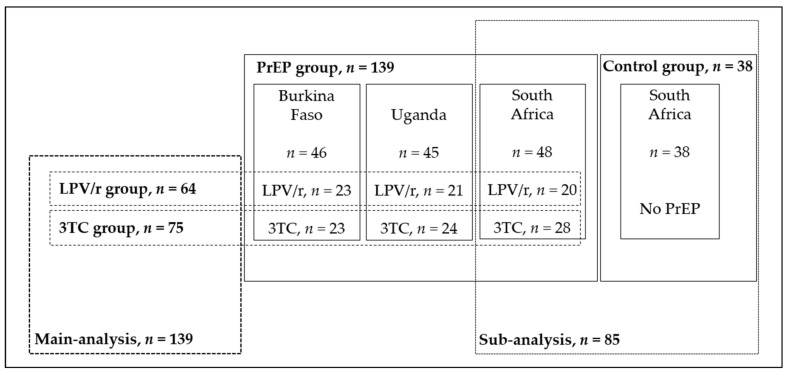
Details of the distribution of final populations analysed by country, group and treatment. LPV/r, lopinavir/ritonavir; 3TC, lamivudine; PrEP, pre-exposure prophylaxis.

After quality control assessment and mtDNA confounds (see Supplementary Materials), a total of 139 CHEU were included for further analysis: 46 children from Burkina Faso, 48 from South Africa, and 45 from Uganda (Figure 2).

At day 7, the mean infant weight was 3.2 ± 0.5 kg (Table 1). Most newborns were born at term and 23 were born at week 36 of the pregnancy. Only two of them had a gestational age under 36 weeks. The majority of children had normal haemoglobin (93.5%) and alanine aminotransferase (92.8%) concentrations, platelet count (94.8%), and white blood cell count (100.0%).

All of the participating mothers had a CD4 cell count above 350 cells/mm^3^ before delivery, and 66.7% had a controlled HIV viral load at D7 (Table 2). Most of them had received zidovudine as prophylaxis during pregnancy for an average of eight weeks. At week 38 post-partum, viral load was uncontrolled in 61.2% of mothers. 

No differences in infant or maternal characteristics exist between the two arms. The study population characteristics by study site are presented in Supplementary Tables S1–S6.

### 3.2. mtDNA Content and Proportion of Deleted mtDNA at D7

At D7 (baseline), mitochondrial DNA copy number per cell (MCN) measured in the PrEP group (*n* = 139) varied from 103 to 1770 copies/cell with a median of 846 and IQR of 555 to 1082 (Table 3). The median MCN in the LPV/r group was 897 (614; 1135) and those in the 3TC group was 803 (484; 1056) (*p* = 0.08). MCN at baseline differed according to study sites (*p* < 0.01, Kruskal–Wallis test) (Table 3). MCN in South Africa was lower than those observed for Burkina Faso and Uganda which were similar (South Africa versus Burkina Faso, *p* value < 0.01; South Africa versus Uganda, *p* value < 0.01; Burkina Faso versus Uganda, *p* value = 0.34). We did not observed difference in MCN according to the gender (Supplementary Table S7). Spearman’s rank correlation test showed that HIV-related maternal characteristics, i.e., CD4 cell count and viral load at D7, did not correlate with MCN at D7 (CD4: Rho = 0.09, *p* = 0.31, *n* = 122; HIV viral load: Rho = −0.03, *p* = 0.72, *n* = 138). Using the experimental threshold for positivity (see Appendix B), 99.3% of children harboured MDD (Table 3). 

### 3.3. mtDNA Content and Proportion of Deleted mtDNA after One Year of PrEP

After one year of PrEP, the overall level of MCN decreased as compared to the matched baseline D7 value, with a median decrease of MCN of 41.7% and IQR of 12.1 to 64.4 (Table 4). This variation differed according to study sites. The overall median decrease of MCN was similar between groups (40.1% for LPV/r versus 42.2% for 3TC, *p* = 0.35). mtDNA depletion was observed in 58 children (41.7%), 27 (42.2%) in the LPV/r group and 31 (41.3%) in the 3TC group (3TC versus LPV/r: *p* = 0.97) (Table 4). As we observed a trend toward an interaction between the prophylactic treatment and the site on mtDNA depletion (*p* = 0.09), we presented the results according to study sites. MDD was detected in all CHEU at week 50.

### 3.4. Sub-Analysis of MCN and MDD Variations among CHEU from South Africa

To answer whether prophylaxis was responsible for mtDNA depletion and deletion, we performed a sub-analysis including a control group of South African CHEU not receiving prophylaxis (Figure 2). As we reported above, both MCN and MDD were site dependent and, due to the inaccessibility of samples from children who had not received prophylaxis for the three sites, we restricted this analysis to South African CHEU. There were no significant differences in the maternal or children’s characteristics between the control group and the PrEP group at baseline (W6) except for the province of residency, prematurity and breastfeeding practice (Supplementary Table S8). 

Evolution of MCN from W6 to W50 revealed that the decrease of MCN occurred during the first six months of life in CHEU in both the control group and the PrEP group. At baseline, MCN were significantly higher in the PrEP group compared to the control group (Table 5), but the inverse trend was observed at W26. 

The decrease of MCN was greater in the PrEP group with a median of 71.3% [59.0;82.1] compared to the control group median of 47.7% (10.4; 55.9) (Table 6). At W26, mtDNA depletion was observed in 16 children (42.1%) in the control group and in 39 children (83.0%) in the PrEP group (Table 6). The proportion of children with MDD was 86.8% in the control group and 100.0% in the PrEP group.

^a^ Wilcoxon Mann-Whitney test for comparison between “Control group” versus “PrEP group Yes”, “Control group” versus “PrEP group LPV/r” and “Control group” versus “PrEP group 3TC”. Abbreviations: MCN, mitochondrial DNA copy number per cell; W6, week 6; W26, week 26; CHEU, children who are HIV-exposed uninfected; PrEP, pre-exposure prophylaxis; IQR, interquartile range; mtDNA, mitochondrial DNA; LPV/r, lopinavir/ritonavir; 3TC, lamivudine.

Risk factor analysis of mtDNA depletion using multivariable Poisson regression with robust error variance after adjustment for confounders showed that infant prophylaxis was positively associated with mtDNA depletion at W26 with an adjusted prevalence ratio (PR) of 1.63 (95% CI: 1.08–2.45) *p* < 0.02 (Table 7). When the type of prophylaxis was introduced, both LPV/r and 3TC treatment were positively associated with mtDNA depletion with an adjusted PR of 1.75 (95%CI: 1.15–2.68; *p* < 0.01) and of 1.54 (95% CI: 1.00–2.37; *p* = 0.05), respectively. Of note, male gender was also a risk factor of mtDNA depletion with an adjusted PR around 1.39 in the two models (Table 7).

## 4. Discussion

In our study, CHEU receiving LPV/r as prophylaxis for one year had the same mtDNA depletion as those receiving 3TC. The sub-analysis with South African control CHEU strongly suggests that both prophylactic regimens are associated with an increased level of MCN depletion. We did not assess the impact of prophylaxis on MDD because the proportion of children with MDD at birth was already high.

We used a threshold for mtDNA depletion of a 50% decrease at W50 or W26, respective to their baseline value (D7 or W6). Using this threshold, the observed decrease of MCN in the PrEP group was greater than any other reported so far. Studies from CHEU have reported an MCN increase in leukocytes from birth to one or two years of age [44], or an unchanged MCN until two years old in PBMCs [48]. Recently, Ajaykumar et al. observed a mild 5.3% decrease of MCN in PBMCs in more than 100 CHEU at one year of age [52]. It is noteworthy that CHEU from South Africa had the larger decrease of MCN given that samples were collected, stored and extracted according to the same procedures, and all the data was analysed using the same quality controls for DNA integrity and PCR validation. In addition, ARV prophylaxis received during pregnancy as per PMTCT national guidelines does not explain this difference. In the South African sub-analysis, we showed that “receiving a prophylaxis” was a risk factor associated with the mtDNA depletion observed in the first six months of life. Furthermore, South African children receiving LPV/r showed a larger decrease in MCN than those receiving the 3TC, which is compatible with a higher toxic effect of LPV/r to 3TC. The deleterious impact of NRTIs, including 3TC, on MCN is commonly recognized in the literature [32,33,34]. PIs and particularly LPV/r–on the other hand–have not been tested. Thus, LPV/r toxicity was unexpected but could be the result of elevated oxidative stress. Indeed, Taura et al. reported that LPV was the most potent inducer of ROS production in PBMCs among the PIs [38]. This production was recently shown to be an alternative mechanism of NRTI-dependent mitochondrial toxicity [66]. Even if the downstream consequences of the ROS production in this context is not clearly established, mtDNA damage might represent a side effect of LPV/r treatment. It should, however, be mentioned that LPV/r excipients consisting of polypropylene glycol and alcohol might also play a role in the process. Further in vitro studies are necessary to assess this hypothesis. The risk factor analysis showed that male gender was positively associated with mtDNA depletion. This sex-driven response has not been reported yet in CHEU. Animal studies in rats and rodents have shown that ROS production was higher in males than in females in several organs including the liver, brain, skeletal muscles and heart [67]. Rodent brains also exhibited higher oxidative damage in males [67]. Taken together, one could suggest that boys are more vulnerable to mtDNA depletion through increased ROS-induced mtDNA damage. Further studies specifically designed for this question are needed.

In the present study, we used MDD as a qualitative variable for mtDNA deletions because we did not validate the linearity of the technique, only the limit of positivity. The proportion of CHEU in the PrEP group with deleted mtDNA at D7, irrespective of the site, was unexpectedly high. To date, Maagaard et al. reported such high prevalence of MDD (75%) in the blood of HIV-infected adults [68]. To our knowledge, two studies have investigated MDD in CHEU, one including Tanzanian children at birth reported a low frequency of MDD [56], and another including US children aged 18 months did not report MDD [55]. These three studies were not quantitative either and did not indicate the limit of positivity. Although the detection of MDD is not reassuring since such mutations cannot be repaired and may accumulate with age [69], it should be kept in mind that cells have more than hundred mtDNA copies, and both deleted and wild type copies co-exist. As a consequence, the expression of such a mutation is considered as recessive. Additional studies using quantitative assays, like combination of next-generation sequencing and advanced sequence analysis, are clearly needed to conclude on the effects of these exposures.

Detecting mtDNA depletion in CHEU raises concerns about its impact on health. It was previously reported that the decrease of MCN is partially reversible as its rebounds upon drug discontinuation [48,70]. On the other hand, CHEU have many unfavourable health outcomes due to unidentified mechanisms. A long-term follow-up of these children, including growth and neurodevelopment outcomes, might bring new insights on this matter.

This study had several limitations. The initial trial did not include children that were not receiving the prophylaxis. Therefore, we cannot draw conclusions on the level of impact of these two prophylaxis regimens on mitochondrial parameters. The sub-analysis in South Africa did include a control group of CHEU but they originated from different provinces. However, they were highly comparable with South African CHEU from the PrEP group and we were able to match the analysis to the same time points, allowing for an age-matched comparison. We were unable to analyse all the MCN corrected by the Hurtado’s formula because we did not record the leucocyte and platelet counts at W50 in the database [63]. However, corrected MCN values at D7 were not significantly different from the raw values. Finally, the maternal HIV viral loads at the time of collection were not available and could not be included in the risk factors analysis.

Our study offers several strengths. First, the random selection of these samples among the participants randomised in the ANRS 12,174 trial minimizes the risk of any selection bias. Adherence to the LPV/r or 3TC was also well monitored and controlled throughout the trial. This group of children not infected but receiving ARV prophylaxis for one year is unique. Our study design compared the variation of the MCN for each individual reducing misinterpretations from cross-sectional studies. Furthermore, the different levels of quality checks (DNA integrity using a more stringent rule for removing samples, i.e., 2 SD versus 5 SD, corrected MCN taking into account platelet and leucocyte counts using the formula of Hurtado et al. [63], and correlation analyses between platelet count and MCN) have already been used and published by others who have worked on the general population or key populations, i.e., drug users [62,71]. Finally, our study’s large sample size ensures powerful analysis of data.

## 5. Conclusions

Mitochondrial depletion was detected in CHEU receiving 3TC but also LPV/r, an antiprotease inhibitor. This class of molecule has never been associated with mitochondrial toxicity, so far. HIV and/or ARV exposures during pregnancy are clearly the prime candidates to explain the unexpectedly high prevalence of children with deleted mtDNA. Long term follow-up of CHEU analysing these outcomes is required as such mutations may accumulate with age.

## Figures and Tables

**Table 1:** Children’s characteristics at PrEP randomisation (D7).

Characteristic	LPV/r (*n* = 64)	3TC (*n* = 75)	Total (*n* = 139)	*p* Value ^a^
Gender; *n* (%)				0.96
Male	33 (51.6)	39 (52.0)	72 (51.8)	
Weight (kg); mean ± SD	3.1 ± 0.5	3.3 ± 0.4	3.2 ± 0.5	0.19
Height (cm); mean ± SD	49.2 ± 2.1	49.8 ± 2.0	49.5 ± 2.0	0.11
Gestational age (week); mean ± SD	38.3 ± 1.7	38.5 ± 1.9	38.4 ± 1.8	0.30
Preterm birth (week); *n* (%)				0.82
No prematurity ≥ 37	53 (82.8)	61 (81.3)	114 (82.0)	
Prematurity < 37	11 (17.2)	14 (18.7)	25 (18.0)	
Hæmoglobin (g/dL); median [IQR]	16.1 [15.0;17.3]	16.1 [14.6;17.3] †	16.1 [14.9;17.3] †	0.91
ALT (U/L); *n* (%)				0.88
Normal ≤ 40	59 (92.2)	70 (93.3)	129 (92.8)	
Abnormal > 40	5 (7.8)	5 (6.7)	10 (7.2)	
Mild [40;100]	3 (4.7)	4 (5.3)	7 (5.0)	
Moderate [100;200]	2 (3.1)	1 (1.3)	3 (2.2)	
Haemoglobin (g/dL); *n* (%)				0.10
Normal > 13	62 (96.9)	67 (90.5) †	129 (93.5) †	
Anemia ≤ 13	2 (3.1)	7 (9.5) †	9 (6.5) †	
Mild [12;13]	1 (1.6)	6 (8.1) †	7 (5.1) †	
Moderate [10;12]	-	1 (1.4) †	1 (0.72) †	
Very severe [0;9]	1 (1.6)	-	1 (0.72) †	
Platelet count (10^3^/mm^3^); *n* (%)				0.43
Normal ≥125	59 (92.2)	69 (97.2) ‡	128 (94.8) ‡	
Thrombocytopenia <125	5 (7.8)	2 (2.8) ‡	7 (5.2) ‡	
Mild [100;125]	3 (4.7)	2 (2.8) ‡	5 (3.7) ‡	
Moderate [50;100]	1 (1.6)	-	1 (0.7) ‡	
Very severe [0;25]	1 (1.6)	-	1 (0.7) ‡	
White cell count (10^3^/mm^3^); *n* (%)				NA
Normal > 2.5	64 (100.0)	73 (100.0) §	137 (100.0) §	
Neutrophil count (10^3^/mm^3^); *n* (%)				0.22
Normal > 1.5	62 (96.9)	73 (100.0) §	135 (98.5) §	
Neutropenia ≤ 1.5	2 (3.1)	-	2 (1.5) §	
Mild [1.25;1.5]	1 (1.6)	-	1 (0.7) §	
Moderate [1.0;1.25]	1 (1.6)	-	1 (0.7) §	

† one missing value, ‡ four missing values, § two missing values. ^a^ Chi-square test or Fisher’s exact test as appropriate and Student’s t-test or Wilcoxon Mann-Whitney test for LPV/r versus 3TC. Abbreviations: PrEP, Pre-exposure prophylaxis; D7, day 7; SD, standard deviation; LPV/r, lopinavir/ritonavir; 3TC, lamivudine; IQR, interquartile range; ALT, Alanine Aminotransferase, NA, non applicable.

**Table 2:** Maternal characteristics at randomisation (D7) and during follow up.

Characteristic	LPV/r (*n* = 64)	3TC (*n* = 75)	Total (*n* = 139)	*p* Value ^a^
At randomisation (D7)				
*Social demographic characteristics*				
Age (year); median [IQR]	27.1 [24.1;30.7]]	28.2 [24.2;32.6]	27.4 [24.1;32.2]	0.25
Parity; median [IQR]	3.0 [2.0;3.5]	2.0 [1.0;4.0]	3.0 [2.0;4.0]	0.56
*Clinical and biological characteristics*				
BMI; median [IQR]	24.0 [21.4;27.5]	23.4 [21.4;27.7]	23.8 [21.4;27.7]	0.88
CD4 cells count (cells/mm^3^); median [IQR]	556.0 [407.0;784.0] ^†^	584 [433.0;760] ^‡^	558.0 [420.0;766.0] ^§^	0.91
HIV viral load (Log copies/mL); median [IQR]	2.2 [2.2;3.3]	2.2 [2.2;3.3] ^¶^	2.2 [2.2;3.3] ^¶^	0.79
HIV viral load control; *n* (%)				0.33
Controlled <1000 copies/mL	40 (62.5)	52 (70.3) ^¶^	92 (66.7) ^¶^	
Uncontrolled ≥1000 copies/mL	24 (37.5)	22 (29.7) ^¶^	46 (33.3) ^¶^	
WHO HIV staging; *n* (%)				1.00
Stage 1	60 (93.8)	71 (94.7)	131 (94.2)	
Stage 2	4 (6.2)	4 (5.3)	8 (5.8)	
*Antiretroviral prophylaxis*				
ARV prophylaxis during pregnancy; *n* (%)				0.47
ZDV	45 (70.3)	51 (68.0)	96 (69.1)	
ZDV + 3TC	11 (17.2)	18 (24.0)	29 (20.9)	
No ARV prophylaxis	8 (12.5)	6 (8.0)	14 (10.1)	
Duration of ARV prophylaxis taken during pregnancy (week); median [IQR]	8.0 [5.5;12.5] ⱡ	8.0 [4.0;14.0] ^¤^	8.0 [5.0;13.0] ^£^	0.63
**During the trial**				
HIV viral load at W38 (Log copies/mL); median [IQR]	3.7 [2.2;4.8]	3.4 [2.2;4.5]	3.5 [2.2;4.7]	0.23
HIV viral load control at W38; *n* (%)				0.18
Controlled < 1000 copies/mL	21 (32.8)	33 (44.0)	54 (38.9)	
Uncontrolled ≥ 1000 copies/mL	43 (67.2)	42 (56.0)	85 (61.2)	
Duration of breastfeeding (week); median [IQR]	45.6 [38.8;48.8]	46.0 [35.7;48.6]	45.9 [37.6;48.1]	0.95

† seven missing values, ‡ ten missing values, § seventeen missing values, ¶ one missing value, ⱡ eight missing values, ¤ six missing values, £ fourteen missing values. ^a^ Chi-square test or Fisher’s exact test as appropriate and Wilcoxon Mann-Whitney test for LPV/r versus 3TC.Abbreviations: D7, day 7; LPV/r, lopinavir/ritonavir; 3TC, lamivudine; IQR, interquartile range; BMI, body mass index; HIV, Human immunodeficiency virus; WHO, World Health Organization; ARV, antiretroviral; ZDV, zidovudine; W38, week 38.

**Table 3:** mtDNA copy number per cell (MCN) and proportion of children with deleted mtDNA (MDD) at PrEP randomisation (D7) by study site.

Site	PrEP	*n*	MCN Median [IQR]	*n*	Children with MDD, *n* (%)
Burkina Faso	All	46	922 [700;1112]	44	43 (97.7)
	LPV/r	23	947 [757;1253]	23	22 (95.7)
	3TC	23	833 [671;1112]	21	21 (100.0)
South Africa	All	48	473 [329;799]	48	48 (100.0)
	LPV/r	20	624 [447;1191]	20	20 (100.0)
	3TC	28	422 [273;715]	28	28 (100.0)
Uganda	All	45	976 [814;1099]	45	45 (100.0)
	LPV/r	21	975 [814;1083]	21	21 (100.0)
	3TC	24	1002 [819;1100]	24	24 (100.0)
All	All	139	846 [555;1082]	137	136 (99.3)
	LPV/r	64	897 [614;1135]	64	63 (98.4)
	3TC	75	803 [484;1056]	73	73 (100.0)

Abbreviations: PrEP, pre-exposure prophylaxis; MCN, mitochondrial DNA copy number per cell; MDD, deleted mitochondrial DNA; D7, day 7; IQR, interquartile range; LPV/r, lopinavir/ritonavir; 3TC, lamivudine.

**Table 4:** Variation of mtDNA copy number per cell (MCN) and number of children with mtDNA depletion (≥50% reduction) after one year of PrEP by study site.

Site	PrEP	*n*	Decrease of MCN Median [IQR]	*p* Value ^a^	Children with Depletion, *n* (%)	*p* Value ^b^
Burkina Faso	All	46	30.2% [2.5;54.4]		13 (28.3)	
	LPV/r	23	39.3% [12.1;65.9]	0.12	8 (34.8)	0.33
	3TC	23	17.3% [−7.7;49.8]		5 (21.7)	
South Africa	All	48	63.3% [37.8;75.0]		30 (62.5)	
	LPV/r	20	73.2% [50.9;85.6]	0.02	15 (75.0)	0.13
	3TC	28	52.8% [34.8;70.4]		15 (53.5)	
Uganda	All	45	35.4% [4.8;52.9]		15 (33.3)	
	LPV/r	21	17.8% [0.4;43.1]	0.08	4 (19.0)	0.06
	3TC	24	49.2% [21.1;59.3]		11 (45.8)	
All	All	139	41.7% [12.1;64.4]		58 (41.7)	
	LPV/r	64	40.1% [14.6;72.7]	0.35	27 (42.2)	0.97
	3TC	75	42.2% [6.9;60.2]		31 (41.3)	

^a^ Wilcoxon Mann-Whitney test for LPV/r versus 3TC. ^b^ Chi square test for LPV/r versus 3TC. Abbreviations: mtDNA, mitochondrial DNA; MCN, mitochondrial DNA copy number per cell; PrEP, pre-exposure prophylaxis; IQR, interquartile range; LPV/r, lopinavir/ritonavir; 3TC, lamivudine.

**Table 5:** mtDNA copy number per cell (MCN) at baseline (W6) and at W26 in South African CHEU from the control group and the PrEP group.

Group	PrEP	*n*	W6 MCN Median [IQR]	*p* Value ^a^	W26 MCN Median [IQR]	*p* Value ^a^
Control	No	38	347 [258;407]		194 [167;248]	
PrEP	Yes	47	458 [310;796]	<0.01	138 [122;148]	<0.01
	LPV/r	19	682 [364;1220]	<0.01	132 [123;143]	<0.01
	3TC	28	422 [273;691]	0.07	141 [122;148]	<0.01

^a^ Wilcoxon Mann-Whitney test for comparison between “Control group” versus “PrEP group”, “Control group” versus “PrEP group LPV/r” and “Control group” versus “PrEP group 3TC”. Abbreviations: MCN, mitochondrial DNA copy number per cell; W6, week 6; W26, week 26; CHEU, children who are HIV-exposed uninfected; PrEP, pre-exposure prophylaxis; IQR, interquartile range; mtDNA, mitochondrial DNA; LPV/r, lopinavir/ritonavir; 3TC, lamivudine.

**Table 6:** Decrease of MCN between baseline (W6) and W26 and mtDNA depletion (≥50% reduction) in South African CHEU from the control group and the PrEP group.

Group	PrEP	*n*	Decrease of MCN Median [IQR]	*p* Value ^a^	Children with mtDNA Depletion, *n* (%)
Control	No	38	47.7% [10.4;55.9]		16 (42.0)
PrEP	Yes	47	71.3% [59.0;88.1]	<0.01	39 (83.0)
	LPV/r	19	79.4% [61.8;88.2]	<0.01	17 (89.5)
	3TC	28	70.5% [55.3;78.9]	<0.01	22 (78.6)

**Table 7:** Risk factors associated with mtDNA depletion (≥50% reduction) after six months of PrEP in CHEU from South Africa.

Independent Variable	Crude Estimates			Adjusted Estimates		
	PR	95% CI (LB-UB)	*p* Value	PR	95% CI (LB-UB)	*p* Value
Gender						
Girl	1.0			1.00		
Boy	1.42	1.03–1.97	0.03	1.39	1.05–1.84	0.02
PrEP *						
No	1.0			1.00		
Yes	1.97	1.33–2.92	<0.01	1.63	1.08–2.45	0.02
Mother age (per 5 years)	1.05	0.92–1.19	0.47			
Parity	0.95	0.81–1.12	0.56			
Duration of breastfeeding (weeks)						
<39	1.00					
≥39	0.85	0.63–1.15	0.30			
Gestational age (weeks)	1.05	0.98–1.13	0.17	1.08	0.98–1.19	0.11
Weight of the infant at W6 (per 500 g)	0.98	0.91–1.07	0.69			

* “PrEP No” refers to the control group and “PrEP Yes” refers to the PrEP group. Abbreviations: mtDNA, mitochondrial DNA; PrEP, pre-exposure prophylaxis; CHEU, children who are HIV-exposed uninfected; PR, prevalence ratio; CI, confidence interval; LB, lower bound; UB, upper bound; W6, week 6.

## Supplementary Materials

The following are available online at https://www.mdpi.com/2077-0383/9/9/2972/s1, Results details: Quality control of DNA; Figure S1: Descriptive statistics of the mitochondrial DNA deletion assay, Figure S2: Intra- and inter-run variations of the mitochondrial DNA deletion assay; Table S1: Children’ characteristics at PrEP randomisation (day 7) in Burkina Faso, Table S2: Maternal characteristics at randomisation (day 7) and during follow up in Burkina Faso, Table S3: Children’ characteristics at PrEP randomisation (day 7) in South Africa, Table S4: Maternal characteristics at randomisation (day 7) and during follow up in South Africa, Table S5: Children’ characteristics at PrEP randomisation (day 7) in Uganda, Table S6: Maternal characteristics at randomisation (day 7) and during follow up in Uganda, Table S7: mtDNA content at randomisation (D7) according to the gender, Table S8: Description of children’ and maternal characteristics in South Africa.

## Appendix A. Methodology Details for Mitochondrial DNA Parameters Assay

### Appendix A.1. Mitochondrial DNA Copy Number Per Cell

The interplate mean of variation coefficients were 4.0% at D7, 3.0% at W6, 3.0% at W26 and 5.0% at W50. Mean Ct for U1A at D7, W6, W26 and W50 were 27.3 ± 2.3, 25.7 ± 0.8, 25.2 ± 0.7 and 27.0 ± 2.6, respectively.

### Appendix A.2. Mitochondrial DNA Deletion

The performance of the assay was validated after a repetitive series of qPCR (*n* = 16) on a single whole blood extracted-DNA whose known concentration ranged from 0.1 ng to 100 ng per assay. Intra- and inter-run variations were 1.8% and 2.65% for MITO, respectively and 1.5% and 2.39% for DEL, respectively (Supplementary Figures S1 and S2).

The efficiency of each individual qPCR was calculated on DNA extracted from platelets cell pellets without nuclear DNA (Etablissement français du Sang, Montpellier, France). DNA was extracted using QIAamp DNA Blood Mini Kit (Qiagen, Hilden, Germany) with RNase treatment and standard curves were constructed for the two pairs of primers using mtDNA whose concentration ranged from 104 to 108 ng/µL.

For the calculation of the proportion of deleted mtDNA (MDD, in %), MITO and DEL experimental values were corrected for their respective qPCR efficiency. We then calculated the proportion of MDD using Equation (3):

(3) $$\mathrm{MDD}\;=\;\frac{corrected\;MITO\;value-corrected\;DEL\;value}{corrected\;MITO\;value}*100$$

## Appendix B. Methodology Details for the Validation of qPCR

The accuracy of the quantification methods was further validated by means of various DNA samples from Rho0 cells, from patients carrying mtDNA deletions, and dried blood from healthy newborns provided by a reference center for mitochondrial diseases of Angers (France). Briefly, qPCR assaying MCN were performed on Rho0 cells which lack mtDNA and which are routinely used as negative PCR quality control for clinical test developments. A faint amplification of mtDNA was observed, but was a result of amplification of known ancestral mtDNA fragments inserted in the Human nuclear genome, also known as NUMTS [72]. After comparing the difference in copy number of this pseudogene (i.e., one copy per cell) versus mtDNA copies (i.e hundreds per cell), we considered this amplification negligible and validated our qPCR. Regarding the mtDNA deletion assay, the co-amplification of the nuclear pseudogene generated an estimated error of 1%. qPCR revealed rates of deleted mtDNA in the blood from patients affected by such mtDNA deletions (*n* = 3) consistent with those observed by the French national reference center. DBS from healthy newborns (*n* = 10) were also tested with our qPCR and showed an average rate of deletion of 1.1% ± 0.4, consistent with the error previously calculated. The assay was qualitative and children were considered positive if the MDD was superior or equal to a threshold of 2.3%, corresponding to mean + 3 *standard deviation, obtained from these healthy newborns.
