# Supplementary material for: Mitochondrial DNA Parameters in Blood of Infants Receiving Lopinavir/Ritonavir or Lamivudine Prophylaxis to Prevent Breastfeeding Transmission of HIV-1
Source: J Clin Med. 2020 Sep 14;9(9):2972. doi: 10.3390/jcm9092972 (PMC7564660; doi:10.3390/jcm9092972)
Supplement: Supplementary file 1 [file jcm-09-02972-s001.pdf]

Results details: Quality control of DNA

In the PrEP group, sixteen out of the 200 CHEU initially selected were excluded from the analyses because they failed of quality control after checking for nuclear DNA degradation at D7 and W50 (seven at D7 and nine at W50). In the sub-analysis, one South African child from the PrEP group (LPV/r arm) was also removed after checking for DNA degradation at W6 and W26.

In the main analysis, Spearman's rank-order correlation test revealed that platelet count did not influence MCN at any point in time, except for in Zambia at D7. In fact, Rho correlation coefficients at D7 were 0.06 for Burkina Faso (p = 0.71), 0.01 for Uganda (p = 0.97), -0.06 for South Africa (p = 0.69) and 0.37 (p = 0.01) for Zambia. At W50, Rho correlation coefficients were 0.04 for Burkina Faso (p = 0.81), -0.004 for South Africa (p = 0.98), 0.27 for Uganda (p = 0.08) and 0.18 for Zambia (p = 0.25). It was previously reported that altitude increased MCN and Zambia has the highest elevation site in the study (1279 meters above sea level) [1, 2]. Therefore, because the experimental design of our study was not applicable to Zambian samples, we ruled this site out from the analyses, leaving a total of 139 CHEU. In the sub-analysis, Rho correlation coefficients were -0.11 (n = 45, 2 missing platelet count values, p = 0.48) at W6 and 0.21 (n = 45, 2 missing platelet count values, p = 0.16) at W26.

We further validated that platelet count did not influence MCN by applying the formula described by Hurtado et al. on the value at D7 [3]. These values did not differ from the raw values. We obtained an overall median difference of 3.1% and an interquartile range (IQR) of 1.96 to 4.71.

Reference

1. Prabhakar, A.; Chatterjee, T.; Bajaj, N.; Tyagi, T.; Sahu, A.; Gupta, N.; Kumari, B.; Nair, V.; Kumar, B.; Ashraf, M.Z. Venous thrombosis at altitude presents with distinct biochemical profiles: a comparative study from the Himalayas to the plains. *Blood Adv* **2019**, *3*, 3713–3723, doi:10.1182/bloodadvances.2018024554.
2. Hui, L.; Rong, W.; Zheng-Ping, J.; Juan, X.; Hua, X. Effects of High Altitude Exposure on Physiology and Pharmacokinetics. *Curr Drug Metab* **2016**, *17*, 559–565, doi:10.2174/1389200216666151015113948.
3. Hurtado-Roca, Y.; Ledesma, M.; Gonzalez-Lazaro, M.; Moreno-Loshuertos, R.; Fernandez-Silva, P.; Enriquez, J.A.; Laclaustra, M. Adjusting MtDNA quantification in whole blood for peripheral blood platelet and leukocyte counts. *PLoS ONE* **2016**, *11*, e0163770, doi:10.1371/journal.pone.0163770.

Supplementary Figures

A

Primers	DNA (ng)	Mean Ct (n = 16)	Standard Deviation	95% CI (Lower Bound)	95% CI (Upper Bound)
MITO	0.1	30.12	1.59	29.34	30.89
	0.5	28.04	1.11	27.49	28.49
	1	27.07	0.63	26.76	27.38
	5	24.86	0.45	24.64	25.08
	10	23.91	0.39	23.71	24.10
	50	21.56	0.17	21.48	21.65
	100	20.26	0.56	19.98	20.53
DEL	0.1	28.84	1.40	28.40	29.28
	0.5	27.06	0.82	26.59	27.53
	1	26.52	0.44	26.10	26.94
	5	23.65	0.26	23.42	23.89
	10	22.70	0.18	22.53	22.86
	50	20.31	0.16	20.20	20.41
	100	18.93	0.18	18.71	19.15

B

Primers	Parameters	Mean	Standard Deviation	95% CI (Lower Bound)	95% CI (Upper Bound)
MITO	Slope	-3.14	0.22	-3.62	-3.20
	Efficiency	1.97	0.08	1.89	2.05
	Y-intercept	27.12	0.62	26.51	27.73
	R^2	0.93	0.02	0.91	0.96
DEL	Slope	-3.71	0.16	-3.87	-3.55
	Efficiency	1.86	0.05	1.81	1.92
	Y-intercept	25.99	0.46	25.54	26.44
	R^2	0.96	0.02	0.94	0.98

Figure S1. Descriptive statistics of the mitochondrial DNA deletion assay. (**A**) Descriptive statistics for sixteen standard replicate analysed on four independent runs (**B**) Descriptive statistics for the qPCR: slope, efficiency, Y-intercept and R^2. Abbreviations: Ct, cycle threshold; CI, confidence interval.

	DNA (ng)	MITO			DEL		
		**Mean Ct**	**Standard Deviation**	**%CV**	**Mean Ct**	**Standard Deviation**	**%CV**
Reproductibility	0.1	30.12	1.59	5.27	28.84	0.90	3.12
	0.5	28.04	1.11	3.97	27.06	0.96	3.55
	1	27.07	0.63	2.34	26.52	0.85	3.21
	5	24.86	0.45	1.79	23.65	0.48	2.03
	10	23.91	0.39	1.63	22.70	0.33	1.45
	50	21.56	0.17	0.79	20.31	0.21	1.03
	100	20.26	0.56	2.78	18.93	0.44	2.32
			2.65%			2.39%	
Repeatability	0.1	30.12	1.40	4.66	28.84	0.80	2.76
	0.5	28.04	0.82	2.93	27.06	0.64	2.37
	1	27.07	0.44	1.62	26.52	0.60	2.25
	5	24.86	0.26	1.06	23.65	0.29	1.22
	10	23.91	0.18	0.73	22.70	0.20	0.89
	50	21.56	0.16	0.74	20.31	0.08	0.39
	100	20.26	0.18	0.88	18.93	0.10	0.54
			1.80%			1.49%	

Figure S2. Intra- and inter-run variations of the mitochondrial DNA deletion assay. Reproducibility was estimated for 16 replicates and repeatability for 4 replicates of 4 independent runs. Abbreviations: Ct, cycle threshold; CV, coefficient of variation.

Supplementary Tables

Table S1. Children's characteristics at PrEP randomisation (D7) in Burkina Faso.

Characteristic	LPV/r (*n* = 23)	3TC (*n* = 23)	Total (*n* = 46)	*p* Value[a]
Gender; *n* (%)				0.76
Male	13 (56.5)	14 (60.9)	27 (58.7)	
Weight (kg); mean ± SD	2.99 ± 0.37	3.02 ± 0.39	3.00 ± 0.36	0.76
Height (cm); mean ± SD	49.2 ± 1.8	49.1 ± 2.2	49.2 ± 2.0	0.88
Gestational age (week); mean ± SD	37.5 ± 2.0	37.7 ± 2.1	37.6 ± 2.0	0.83
Preterm birth (week); *n* (%)				0.37
No prematurity ≥37	15 (65.2)	12 (52.2)	27 (58.7)	
Prematurity <37	8 (37.8)	11 (47.8)	19 (41.3)	
Hæmoglobin (g/dL); median [IQR]	15.2 [14.2;17.2]	15.6 [14.0;17.2]	15.3 [14.2;17.2]	0.95
ALT (U/L); *n* (%)				0.35
Normal ≤40	19 (82.6)	22 (95.7)	41 (89.1)	
Abnormal >40	4 (17.4)	1 (4.3)	5 (10.9)	
Mild]40;100]	2 (8.7)	1 (4.3)	-	
Moderate]100;200]	2 (8.7)	-	-	
Hæmoglobin (g/dL); *n* (%)				0.23
Normal >13	23 (100.0)	20 (87.0)	43 (93.5)	
Anemia ≤13	-	3 (13.0)	3 (6.5)	
Mild [12;13]	-	3 (13.0)	-	
Platelet count ($10^3/mm^3$); *n* (%)				1.00
Normal ≥125	22 (95.7)	-	45 (97.8)	
Thrombocytopenia <125	1 (4.3)	-	1 (2.2)	
Mild [100;125[	1 (4.3)	-	1 (2.2)	
White cell count ($10^3/mm^3$); *n* (%)				NA
Normal >2.5	23 (100.0)	23 (100.0)	46 (100.0)	
Neutrophil count ($10^3/mm^3$); *n* (%)				NA
Normal >1.5	23 (100.0)	23 (100.0)	46 (100.0)	

[a]Chi-square test or Fisher's exact test as appropriate and Student's t-test or Wilcoxon Mann-Whitney test for LPV/r versus 3TC. Abbreviations: PrEP, Pre-exposure prophylaxis; D7, day 7; SD, standard deviation; LPV/r, lopinavir/ritonavir; 3TC, lamivudine; IQR, interquartile range; ALT Alanine Aminotransferase, NA non applicable.

Table S2. Maternal characteristics at randomisation (D7) and during follow up in Burkina Faso.

Characteristic	LPV/r (*n* = 23)	3TC (*n* = 23)	Total (*n* = 46)	*p* Value [a]
**At randomisation**				
Social demographic characteristics				
Age (year); median [IQR]	26.8 [25.0;31.8]	28.2 [25.0;34.2]	28.0 [25.0;33.0]	0.23
Parity; median [IQR]	3.0 [2.0;4.0]	3.0 [2.0;5.0]	3.0 [2.0;4.0]	0.32
Clinical and biological characteristics				
BMI; median [IQR]	23.0 [20.7;24.2]	22.1 [21.1;24.5]	22.5 [21.0;24.2]	0.93
CD4 cells count (cells/mm^3); median [IQR]	706.5 [492.5;839.0][†]	604.0 [489.0;729.0][‡]	617.0 [489.0;811.0][§]	0.56
HIV viral load (Log copies/mL); median [IQR]	2.9 [2.2;3.5]	2.2 [2.2;3.0]	2.6 [2.2;3.5]	0.28
HIV viral load control; *n* (%)				0.12
Controlled <1000 copies/mL	12 (52.2)	18 (78.3)	30 (65.2)	
Uncontrolled ≥1000 copies/mL	11 (47.8)	5 (21.7)	16 (34.8)	
WHO HIV staging; *n* (%)				1.00
Stage 1	21 (91.3)	21 (91.3)	42 (91.3)	
Stage 2	2 (8.7)	2 (8.7)	4 (8.7)	
Antiretroviral prophylaxis				

ARV prophylaxis during pregnancy; *n* (%)				1.00
ZDV	22 (95.7)	22 (95.7)	44 (95.7)	
No ARV prophylaxis	1 (4.3)	1 (4.3)	2 (4.3)	
Duration of ARV prophylaxis taken during pregnancy (week); median [IQR]	7.5 [5.0;10.0]	6.5 [4.0;10.0]	7.5 [5.0;10.0]	0.83
**During the trial**				
HIV viral load at W38 (Log copies/mL); median [IQR]	4.7 [3.4;5.5]	4.5 [2.2;5.2]	4.6 [3.2;5.3]	0.42
HIV viral load control at W38; *n* (%)				0.72
Controlled <1000 copies/mL	4 (17.4)	6 (26.1)	10 (21.7)	
Uncontrolled ≥1000 copies/mL	19 (82.6)	17 (73.9)	36 (78.3)	
Duration of breastfeeding (week); median [IQR]	47.9 [46.1;49.7]	47.4 [46.6;50.6]	47.7 [46.6;49.7]	0.94

† three missing values, ‡ four missing values, § seven missing values. [a] Chi-square test or Fisher's exact test as appropriate and Wilcoxon Mann-Whitney test for LPV/r versus 3TC. Abbreviations: D7, day 7; LPV/r, lopinavir/ritonavir; 3TC, lamivudine; IQR, interquartile range; BMI, body mass index; HIV, Human immunodeficiency virus; WHO, World Health Organization; ARV, antiretroviral; ZDV, zidovudine; W38, week 38.

Table S3. Children's characteristics at PrEP randomisation (D7) in South Africa.

Characteristic	LPV/r (*n* = 20)	3TC (*n* = 28)	Total (*n* = 48)	*p* Value [a]
Gender; *n* (%)				0.41
Male	11 (55.0)	12 (42.9)	23 (48.0)	
Weight (kg); mean ± SD	3.2 ± 0.5	3.4 ± 0.4	3.3 ± 0.5	0.18
Height (cm); mean ± SD	48.5 ± 1.7	49.5 ± 1.9	49.1 ± 1.9	0.06
Gestational age (week); mean ± SD	37.8 ± 1.0	38.0 ± 1.5	37.9 ± 1.3	0.67
Preterm birth (week); *n* (%)				0.68
No prematurity ≥37	17 (85.0)	25 (89.3)	42 (87.5)	
Prematurity <37	3 (15.0)	3 (10.7)	6 (12.5)	
Hæmoglobin (g/dL); median [IQR]	16.5 [15.8;18.2]	17.1 [14.3;19.0]†	16.9 [15.1;18.7]†	0.81
ALT (U/L); *n* (%)				1.00
Normal ≤40	19 (95.0)	26 (92.9)	45 (93.8)	
Abnormal >40	1 (5.0)	2 (7.1)	3 (6.2)	
Mild]40;100]	1 (5.0)	1 (3.6)	2 (4.2)	
Moderate]100;200]	-	1 (3.6)	1 (2.1)	
Hæmoglobin (g/dL); *n* (%)				0.84
Normal >13	18 (90.0)	25 (92.6)†	43 (91.5)†	
Anemia ≤13	2 (10.0)	2 (7.4)†	4 (8.5)†	
Mild [12;13]	1 (5.0)	1 (3.7)†	2 (4.3)†	
Moderate [10;12[	-	1 (3.7)†	1 (2.1)†	
Very severe [0;9[	1 (5.0)	-	1 (2.1)†	
Platelet count (10^3/mm^3); *n* (%)				0.46
Normal ≥125	16 (80.0)	22 (91.7)‡	38 (86.4)‡	
Thrombocytopenia <125	4 (20.0)	2 (8.3)‡	6 (13.6)‡	
Mild [100;125[	2 (10.0)	2 (8.3)‡	4 (9.1)‡	
Moderate [50;100[	1 (5.0)	-	1 (2.3)‡	
Very severe [0;25[	1 (5.0)	-	1 (2.3)‡	
White cell count (10^3/mm^3); *n* (%)				NA
Normal >2.5	20 (100.0)	26 (100.0)§	46 (100.0)§	
Neutrophil count (10^3/mm^3); *n* (%)				NA
Normal >1.5	20 (100.0)	26 (100.0)§	46 (100.0)§	

† one missing value, ‡ four missing values, § two missing values. [a]Chi-square test or Fisher's exact test as appropriate and Student's t-test or Wilcoxon Mann-Whitney test for LPV/r versus 3TC. Abbreviations:

PrEP, Pre-exposure prophylaxis; D7, day 7; SD, standard deviation; LPV/r, lopinavir/ritonavir; 3TC, lamivudine; IQR, interquartile range; ALT Alanine Aminotransferase, NA non applicable.

Table S4. Maternal characteristics at randomisation (D7) and during follow up in South Africa.

Characteristic	LPV/r (*n* = 20)	3TC (*n* = 28)	Total (*n* = 48)	*p* value[a]
**At randomisation**				
Social demographic characteristics				
Age (year); median [IQR]	28.4 [22.4;34.1]	30.0 [24.5;33.9]	29.3 [24.1;34.1]	0.71
Parity; median [IQR]	2.0 [1.0;3.0]	2.0 [1.0;3.0]	2.0 [1.0;3.0]	0.37
Clinical and biological characteristics				
BMI; median [IQR]	28.0 [24.3;33.2]	28.3 [23.4;33.3]	28.0 [24.2;33.2]	0.84
CD4 cells count (cells/mm^3); median [IQR]	420.0 [363.0;672.0]†	529.0 [393.0;805.5]‡	495.0 [389.0;710.0]§	0.30
HIV viral load (Log copies/mL); median [IQR]	2.2 [2.2;3.5]	2.5 [2.2;3.5]	2.2 [2.2;3.5]	0.83
HIV viral load control; *n* (%)				0.76
Controlled <1000 copies/mL	13 (65.0)	17 (60.7)	30 (62.5)	
Uncontrolled ≥1000 copies/mL	7 (35.0)	11 (39.3)	18 (37.5)	
WHO HIV staging; *n* (%)				NA
Stage 1	20 (100.0)	28 (100.0)	48 (100.0)	
Antiretroviral prophylaxis				
ARV prophylaxis during pregnancy; *n* (%)				NA
ZDV	20 (100.0)	28 (100.0)	48.0 (100.0)	
Duration of ARV prophylaxis taken during pregnancy (week); median [IQR]	13.5 [8.0;19.5]	14.0 [8.5;16.0]	13.5 [8.0;17.0]	0.66
During the trial				
HIV viral load at W38 (Log copies/mL); median [IQR]	3.3 [2.4;4.0]	3.5 [2.8;4.4]	3.4 [2.7;4.2]	0.52
HIV viral load control at W38; *n* (%)				0.76
Controlled <1000 copies/mL	7 (35.0)	11 (39.3)	18 (37.5)	
Uncontrolled ≥1000 copies/mL	13 (65.0)	17 (60.7)	30 (62.5)	
Duration of breastfeeding (week); median [IQR]	36.7 [28.3;48.1]	46.0 [33.4;49.8]	44.7 [31.1;49.4]	0.14

† one missing value, ‡ four missing values, § five missing values. [a] Chi-square test or Fisher's exact test as appropriate and Wilcoxon Mann-Whitney test for LPV/r versus 3TC. Abbreviations: D7, day 7; LPV/r, lopinavir/ritonavir; 3TC, lamivudine; IQR, interquartile range; BMI, body mass index; HIV, Human immunodeficiency virus; WHO, World Health Organization; ARV, antiretroviral; ZDV, zidovudine; W38, week 38.

Table S5. Children's characteristics at PrEP randomisation (D7) in Uganda.

Characteristic	LPV/r (*n* = 21)	3TC (*n* = 24)	Total (*n* = 45)	*p* Value [a]
Gender; *n* (%)				0.45
Male	9 (42.9)	13 (54.2)	22 (48.9)	
Weight (kg); mean ± SD	3.2 ± 0.5	3.4 ± 0.4	3.3 ± 0.5	0.34
Height (cm); mean ± SD	49.9 ± 2.4	50.9 ± 1.6	50.4 ± 2.0	0.10
Gestational age (week); mean ± SD	39.8 ± 1.0	39.6 ± 0.8	39.9 ± 1.1	0.34
Preterm birth (week); *n* (%)				NA
No prematurity ≥37	21 (100.0)	24 (100.0)	45 (100.0)	
Hæmoglobin (g/dL); median [IQR]	16.6 [15.4;17.2]	16.0 [15.1;17.0]	16.4 [15.2;17.1]	0.37
ALT (U/L); *n* (%)				0.49
Normal ≤40	-	22 (91.7)	43 (95.6)	
Abnormal >40	-	2 (8.3)	2 (4.4)	
Mild]40;100]	-	2 (8.3)	2 (4.4)	
Hæmoglobin (g/dL); *n* (%)				0.49
Normal >13	-	22 (91.7)	43 (95.6)	
Anemia ≤13	-	2 (8.3)	2 (4.4)	
Mild [12;13]	-	2 (8.3)	2 (4.4)	

Platelet count ($10^3/mm^3$); *n* (%)				NA
Normal ≥125	21 (100.0)	24 (100.0)	45 (100.0)	
White cell count ($10^3/mm^3$); *n* (%)				NA
Normal >2.5	21 (100.0)	24 (100.0)	45 (100.0)	
Neutrophil count ($10^3/mm^3$); *n* (%)				0.21
Normal >1.5	19 (90.5)	-	43 (95.6)	
Neutropenia ≤1.5	2 (9.5)	-	2 (4.4)	
Mild [1.25;1.5]	1 (4.8)	-	1 (2.2)	
Moderate [1.0;1.25[	1 (4.8)	-	1 (2.2)	

[a] Chi-square test or Fisher's exact test as appropriate and Student's t-test or Wilcoxon Mann-Whitney test for LPV/r versus 3TC. Abbreviations: PrEP, Pre-exposure prophylaxis; D7, day 7; SD, standard deviation; LPV/r, lopinavir/ritonavir; 3TC, lamivudine; IQR, interquartile range; ALT Alanine Aminotransferase, NA non applicable.

Table S6. Maternal characteristics at randomisation (D7) and during follow up in Uganda.

Characteristic	LPV/r (*n* = 21)	3TC (*n* = 24)	Total (*n* = 45)	*p* Value [a]
**At randomisation**				
Social demographic characteristics				
Age (year); median [IQR]	25.2 [24.1;28.7]	27.6 [23.7;30.7]	26.3 [24.1;30.2]	0.48
Parity; median [IQR]	3.0 [3.0;4.0]	2.0 [2.0;5.5]	3.0 [2.0;4.0]	0.50
Clinical and biological characteristics				
BMI; median [IQR]	22.6 [21.1;24.6]	22.9 [20.9;24.1]	22.8 [21.0;24.4]	0.81
CD4 cells count (cells/mm^3); median [IQR]	578.5 [471.0;858.0]†	536.0 [436.0;786.0]‡	565.5 [442.5;802.5]§	0.44
HIV viral load (Log copies/mL); median [IQR]	2.2 [2.2;3.0]	2.2 [2.2;3.2]¶	2.2 [2.2;3.1]¶	0.83
HIV viral load control; *n* (%)				0.85
Controlled <1000 copies/mL	15 (71.4)	17 (70.8)¶	32 (72.7)¶	
Uncontrolled ≥1000 copies/mL	6 (28.6)	6 (26.1)¶	12 (27.3)¶	
WHO HIV staging; *n* (%)				1.00
Stage 1	19 (90.5)	22 (91.7)	41 (91.1)	
Stage 2	2 (9.5)	2 (8.3)	4 (8.9)	
Antiretroviral prophylaxis				
ARV prophylaxis during pregnancy; *n* (%)				0.29
ZDV	3 (14.3)	1 (4.2)	4 (8.9)	
ZDV + 3TC	11 (52.4)	18 (75.0)	29 (64.4)	
No ARV prophylaxis	7 (33.3)	5 (20.8)	12 (26.7)	
Duration of ARV prophylaxis taken during pregnancy (week); median [IQR]	5.0 [4.0;11.0]	6.0 [4.0;8.0]	6.0 [4.0;8.0]	0.82
**During the trial**				
HIV viral load at W38 (Log copies/mL); median [IQR]	3.0 [2.2;4.4]	2.2 [2.2;3.8]	2.2 [2.2;4.0]	0.08
HIV viral load control at W38; *n* (%)				0.20
Controlled <1000 copies/mL	10 (47.6)	16 (66.7)	26 (57.8)	
Uncontrolled ≥1000 copies/mL	11 (52.4)	8 (33.3)	19 (42.2)	
Duration of breastfeeding (week); median [IQR]	42.4 [39.1;45.4]	37.9 [33.4;42.9]	41.4 [33.4;44.9]	0.14

† three missing values, ‡ two missing values, § five missing values, ¶ one missing value. [a]Chi-square test or Fisher's exact test as appropriate and Wilcoxon Mann-Whitney test for LPV/r versus 3TC. Abbreviations: D7, day 7; LPV/r, lopinavir/ritonavir; 3TC, lamivudine; IQR, interquartile range; BMI, body mass index ; HIV, Human immunodeficiency virus ; WHO, World Health Organization; ARV, antiretroviral; ZDV, zidovudine; W38, week 38.

Table S7. mtDNA content at randomisation (D7) according to the gender.

Site	PrEP	*n*	Female median [IQR]	*n*	Male median [IQR]	*p* Value [a]
Burkina Faso	All	19	895 [700;1128]	27	947 [648;1112]	0.96
	LPV/r	10	897 [811;1253]	13	969 [757;1061]	0.74
	3TC	9	833 [700;1034]	14	866 [648;1112]	1.00
South Africa	All	25	458 [322;782]	23	565 [337;1031]	0.43
	LPV/r	9	566 [458;796]	11	682 [436;1220]	0.82
	3TC	16	422 [244;691]	12	418 [273;762]	0.70
Uganda	All	23	921 [783;1101]	22	1024 [852;1099]	0.45
	LPV/r	12	948 [813;1048]	9	1004 [846;1143]	0.60
	3TC	11	908 [774;1129]	13	1045 [911;1095]	0.65
All	All	67	811 [527;1034]	72	907 [560;1089]	0.29
	LPV/r	31	865 [683;1083]	33	947 [555;1162]	0.72
	3TC	36	761 [439;992]	39	880 [566;1076]	0.27

[a] Wilcoxon Mann-Whitney test.

Table S8. Description of children' and maternal characteristics in South Africa.

Characteristic	Control group (*n* = 38)	PrEP group (*n* = 47)			*p* Value [a]
**Children**					
**At W6**					
		LPV/r (*n* = 19)	3TC (*n* = 28)	Total (*n* = 47)	
Gender; *n* (%)					0.92
Male	19 (50.0)	10 (52.6)	13 (46.4)	23 (48.9)	
Province of South Africa					<0.01
Eastern Cape	6 (15.8)	19 (100.0)	28 (100.0)	47 (100.0)	
Others	32 (84.2)	-	-	-	
Weight (kg); mean ± SD	4.7 ± 1.0[†]	4.5 ± 0.6[†]	4.7 ± 0.6[‡]	4.6 ± 0.6[$]	0.14
Height (cm); mean ± SD	53.1 ± 5.2[¶]	53.1 ± 1.7[†]	54.0 ± 1.9[‡]	53.7 ± 1.8[$]	0.73
Gestational age (week); mean ± SD	37.0 ± 3.0[ł]	37.8 ± 1.0	38.0 ± 1.5	37.9 ± 1.3	0.53
Preterm birth (week); *n*(%)					0.02
No prematurity ≥37	15 (65.2)[ł]	16 (84.2)	26 (92.9)	42 (89.4)	
Prematurity <37	8 (34.8)[ł]	3 (15.8)	2 (7.1)	5 (10.6)	
**Mother**					
**At W6**					
Age (year); median [IQR]	26.0 [23.0;30.0]	29.1 [22.5;34.4]	30.0 [23.5;33.9]	29.5 [23.0;34.4]	0.08
Parity; median [IQR]	2.0 [2.0;3.0]	2.0 [1.0;3.0]	2.0 [1.0;3.0]	2.0 [1.0;3.0]	0.09
**During the study**					
Breastfeeding					<0.01
Yes	21 (75.0)[£]	19 (100.0)	28 (100.0)	47 (100.0)	
No	7 (25.0)[£]	-	-	-	
Duration of breastfeeding; median; *n* (%)					0.82
<39 weeks	10 (35.7)[£]	10 (52.6)	8 (28.6)	18 (38.3)	
≥39 weeks	18 (64.3)[£]	9 (47.4)	20 (71.4)	29 (61.7)	

† five missing values, ‡ one missing value, $ six missing values, ¶ twenty-seven missing values, ł fifteen missing values, £ ten missing values. [a] Wilcoxon Mann-Whitney test and Chi-square test or Fisher's exact test as appropriate for comparison between "Control group" (n = 38) versus "PrEP group" (n = 47). Abbreviations: PrEP, pre-exposure prophylaxis; W6, week 6; LPV/r, lopinavir/ritonavir; 3TC, lamivudine; SD, standard deviation.
